# Effects of Osmotic Dehydration on Mass Transfer of Tender Coconut Kernel

**DOI:** 10.3390/foods13142188

**Published:** 2024-07-11

**Authors:** Sihao Wu, Juntao Wang, Lin Zhang, Sixin Liu, Congfa Li

**Affiliations:** 1 School of Food Science and Engineering, Hainan University, Haikou 570228, China; 2 Key Laboratory of Food Nutrition and Functional Food of Hainan Province, Haikou 570228, China; 3 Key Laboratory of Tropical Agricultural Products Processing Technology of Haikou, Haikou 570228, China

**Keywords:** tender coconut kernel, solid-state osmotic dehydration, liquid-state osmotic dehydration, dehydration, mass transfer

## Abstract

Tender coconut water has been very popular as a natural beverage rich in various electrolytes, amino acids, and vitamins, and hence a large amount of tender coconut kernel is left without efficient utilization. To explore the possibility of making infused tender coconut kernel, we investigated the effects of two osmosis methods, including solid-state osmotic dehydration and liquid-state osmotic dehydration, as well as two osmosis agents such as sorbitol and sucrose, on the mass transfer of coconut kernel under solid-state osmotic dehydration conditions. The results showed that under the conditions of solid-state osmosis using sucrose and liquid-state osmosis using sucrose solution, the water diffusion coefficients were 9.0396 h^−1/2^ and 2.9940 h^−1/2^, respectively, with corresponding water mass transfer coefficients of 0.3373 and 0.2452, and the equilibrium water loss rates of 49.04% and 17.31%, respectively, indicating that the mass transfer efficiency of solid-state osmotic dehydration of tender coconut kernel was significantly higher than that of liquid-state osmotic dehydration. Under solid osmosis conditions, the water loss rates using sucrose and sorbitol were 38.64% and 41.95%, respectively, with dry basis yield increments of 61.38% and 71.09%, respectively, demonstrating superior dehydration efficiency of sorbitol over sucrose under solid-state osmosis. This study can provide a reference for the theoretical study of the mass transfer of tender coconut kernel through osmotic dehydration, and also provide technical support for the development and utilization of tender coconut kernel.

## 1. Introduction

Coconut is a typical tropical crop, and the white and hard kernel in the fruits has high nutritional value and good flavor [1]. It is widely used in the production of coconut oil, coconut milk, candy, biscuits, sugar-infused food, etc. [2]. In Hainan, the main and most important coconut palm-growing province in China, the white kernel from the ripe fruit is commonly processed into sugar-infused coconut. However, sugar infusion is usually very difficult and time-consuming, and the product tastes very hard and rough due to the hard texture of the white kernel.

In the past 20 years, tender coconut water, rich in various electrolytes, amino acids, and vitamins, has gained wide popularity around the world. Therefore, a large amount of tender coconut kernel is left. In Hainan, the coconut kernel is usually wasted, except for a small part, which is used as the main ingredient for “Qingbuliang”, a local specialty snack food in the streets. The tender coconut kernel is soft in texture and has a subtle coconut flavor, which may be a good raw material for a sugar-infused product. However, there is no research report related to the processing and utilization of tender coconut kernels.

Sugar infusion, or osmotic dehydration, is a widely used method for fruit and vegetable processing. Osmotic dehydration, including solid-state osmotic dehydration (SSD) and liquid-state osmotic dehydration (LOD), involves immersing fruits and vegetables in a high-osmotic-pressure solution, such as sugar and salt solution, after pretreatment or directly mixing with a solid osmotic agent, such as sugars and salts. The main factors affecting the osmotic dehydration of fruits and vegetables include osmotic mode, osmotic agent type and concentration, osmotic time, raw material structure, etc. [3]. Sugar, which is the most popular osmotic agent, presents many health risks. Therefore, the use of unconventional natural sweeteners like honey, jaggery, sorbitol, coconut sugar, stevia, and sugar beet molasses as an osmotic agent is of utmost significance. The use of natural sweeteners, viz., jaggery and honey, as osmotic agents has numerous health benefits. Due to these benefits, they can be used at small scales, in home-scale industries, and in large-scale industries [4,5]. The type of osmotic agent strongly affects osmotic dehydration parameters, such as water loss and solute increase. In recent years, there has been increasing interest in reducing sugar consumption, and many studies have focused on the use of polyols to obtain preserves or dried fruit during osmotic dehydration. Wiktor et al. evaluated the effects of polyols (mannitol and sorbitol) at different concentrations on the process dynamics and selected the physical and chemical properties of osmotically dehydrated organic strawberries [6]. Pravitha et al. evaluated the feasibility of unconventional osmotic agents such as coconut sugar and jaggery to produce coconut flakes, compared the quality and sensory properties of coconut flakes treated with sucrose, and found that the introduction of these new osmotic agents had a positive impact on sensory properties [7]. There are also reports on coupled osmosis technology. For example, Seyed-Hassan et al. studied the effects of coupled osmosis dehydration by cold plasma pretreatment on the drying kinetics and quality of *Agariculus bisporus* and found that atmospheric pressure cold plasma pretreatment had a positive effect on mushroom osmosis dehydration [8]. Much research work is reported on the process and mechanism of LOD, such as mango [9,10], kiwifruit [11], blackberry [12], apple [13], and so on. At the macro level, the diffusion coefficients of fruits such as apples, kiwifruit, and pineapple in sucrose solution range from 10^−12^ m^2^/s to 10^−8^ m^2^/s [9,11,14]. However, there are few reports about SSD mass transfer of tender coconut kernels. In particular, the effect of sugar and sugar substitutes as osmotic agents on the SSD of tender coconut kernels has not been reported.

Some models, including empirical models, semi-empirical models, and theoretical models, are proposed to better predict the mass transfer dynamics of the osmotic dehydration process. Empirical and semi-empirical models include the Azuara model, the Page model, etc. [15]. Prithani et al. [11] applied the Azuara model to the process of kiwifruit osmotic dehydration, assisted by ultrasonic technology. The balance value of water loss in the ultrasonic treatment group was 58.47%, 16.52% higher than that in the control group. In terms of theoretical models, the diffusion coefficient, which is defined as the rate of a substance passing through a unit cross-sectional area divided by the concentration gradient on the cross-section, is used to express the diffusion rate in the kinetic equation according to Fick’s second law of diffusion. The diffusion coefficient can be calculated by fitting the test data with the equation, and the rate of material diffusion can be evaluated [16].

In this paper, we will investigate the effects of two osmosis methods, including SSD and LOD, on the mass transfer of tender coconut kernel, as well as two osmosis agents, such as sorbitol and sucrose, on the mass transfer of coconut kernel under solid-state osmotic dehydration conditions. It will provide the theoretical reference and technical support for the sugar infusion of tender coconut kernel.

## 2. Materials and Methods

### 2.1. Tender Coconut Kernel Treatment and Osmotic Dehydration

Tender coconuts, with a ripeness of 80% and a thickness of 5 mm to 8 mm in the white flesh, were purchased from the local market. The coconut kernel was cut into 3 cm × 4 cm cubes and put into the boiling solution containing 0.5% citric acid, with a ratio of 1:3 (coconut kernel to the solution) to protect the coconut kernel color. When the solution was boiling again and kept for 1min, the coconut kernel was taken out, drained, and cooled to the ambient temperature. Then, 100 g of processed coconut cubes was weighed and placed in every flask. 

The methods of different osmosis modes are as follows: For SSD, every flask was added with 100 g sucrose and 0.02 g potassium sorbate (to inhibit possible microbial growth and avoid interfering with the experiment). For LOD, every flask was added with 100 mL 50% sucrose solution and 0.02 g potassium sorbate [17].

The methods of different osmotic agents in SSD are as follows: every flask was added with 100 g sucrose or sorbitol and 0.02 g potassium sorbate.

Each flask was shaken every 15 min. During the process, 100 g of the coconut cubes was taken out at 0.5 h, 1 h, 2 h, 4 h, 8 h, 12 h, 16 h, 20 h, and 24 h, respectively. The surface of the coconut kernel was quickly washed with distilled water, and the water on the surface was sucked up with absorbent paper, and then various indexes were measured [18].

### 2.2. Determination of Moisture Content

The moisture content was measured by the constant weight method according to GB 5009.3-2016 [19]. 

### 2.3. Determination of Soluble Solids

Soluble solids were measured by Abbe refractometer [20].

### 2.4. Calculation of Solid Content Increase Rate (SG), Dry Base Solid Content Increase Rate (DSG), Water Loss Rate (WL), and Dehydration Efficiency (DEI)

SG, DSG, WL, DEI were measured according to the method of Zhang [21].

(1) $$\mathrm{SG}=\frac{M_{t}\cdot X_{st}-M_{0}\cdot X_{s0}}{M_{0}}$$

(2) $$\mathrm{DSG}=\frac{M_{t}\cdot X_{st}-M_{0}\cdot X_{s0}}{M_{0}\cdot X_{s0}}$$

(3) $$\mathrm{WL}=\frac{M_{0}\cdot X_{w0}-M_{t}\cdot X_{wt}}{M_{0}}$$

(4) $$\mathrm{DEI}=\frac{WL}{SG}$$

where t is the osmosis time of the sample (h); M_0_ and M_t_ are the mass (g) at the initial and specified osmosis time t of the sample, respectively; X_s0_ and X_st_ are the solid content (%) at the initial and specified osmosis time t of the sample, respectively; and X_w0_ and X_wt_ are the moisture content (%) at the initial and specified osmosis time t of the sample, respectively.

### 2.5. Calculation of Water Diffusion Coefficient and Solid Diffusion Coefficient of Tender Coconut Kernel

According to the method of Carlos et al. [22], Fick diffusion equation was used to calculate the diffusion coefficient of water and solid matter. The formulae are as follows:

WL = K_W_·(t)^0.5^ + A (5)

SG = K_S_·(t)^0.5^ + B (6)

where K_W_ is the water diffusion coefficient of the sample (h^−1/2^); K_S_ is the solid diffusion coefficient of the sample (h^−1/2^); t is the osmosis time of the sample (h); and A and B are constants.

### 2.6. Calculation of Mass Transfer Coefficient and Osmotic Equilibrium Point of Coconut Kernel 

According to the method of Wei [23], the expressions of WL and SG are as follows:

(7) $$\mathrm{WL}=\frac{S_{1}\cdot t\cdot WL_{\infty }}{1+S_{1}\cdot t}$$

(8) $$\mathrm{SG}=\frac{S_{2}\cdot t\cdot SG_{\infty }}{1+S_{2}\cdot t}$$

The above two equations can be transformed into the following equation:

(9) $$\frac{t}{WL}=\frac{1}{S_{1}\cdot WL_{\infty }}\;+\frac{t}{WL_{\infty }}$$

(10) $$\frac{t}{SG}=\frac{1}{S_{2}\cdot SG_{\infty }}\;+\frac{t}{SG_{\infty }}$$

The values of WL and SG are measured from the experimental data of different osmosis times, and the equilibrium water loss (WL_∞_) and solid gain (SG_∞_) are estimated from the slope and intercept of the curve of (t/WL) and (t/SG) to t.

### 2.7. Statistical Analysis

All the experiments were carried out in triplicate. IBM SPSS Statistics 23.0 software was used to perform a one-way ANOVA on the data, and *p* < 0.05 was considered statistically significant. Origin 2019b software was used to plot the data, and regression analysis was performed on the corresponding data. 

## 3. Results

### 3.1. Effects of SSD and LOD of Sucrose on Dehydration and Mass Transfer of TCK

#### 3.1.1. Effects of SSD and LOD of Sucrose on the Water Loss Rate of TCK

**Figure 1:**
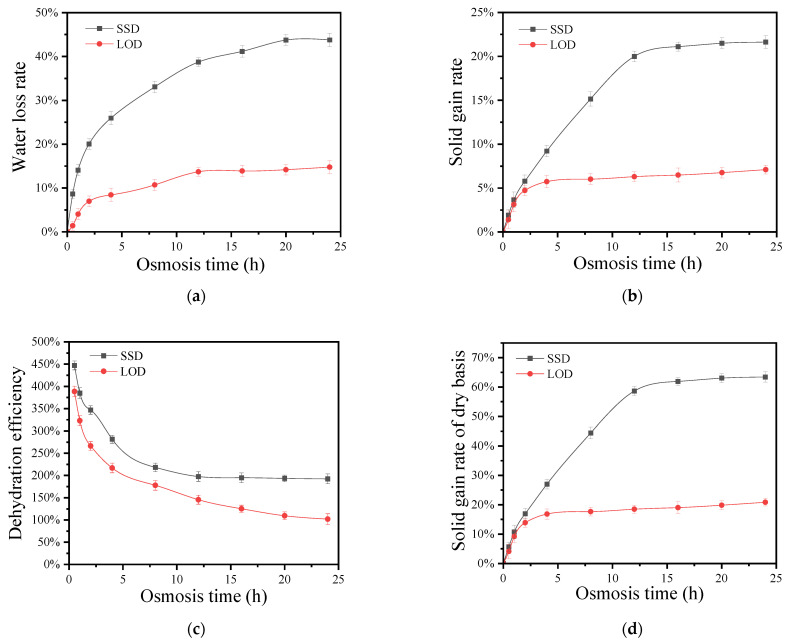
Changes in water loss rate (**a**), solid gain rate (**b**), dry basis solid gain rate (**c**), and dehydration efficiency (**d**) during SSD and LOD of coconut kernel.

In the study of mass transfer by osmotic dehydration, the water loss rate (WL) is defined as the difference in the mass of water in the coconut kernel between the initial time and a specified osmosis time, divided by the initial mass of the coconut kernel. WL quantifies the extent of water loss during the osmosis process. Similarly, the solid gain rate (SG) is calculated as the difference in the mass of solids in the coconut kernel between a specified osmosis time and the initial time, divided by the initial mass of the coconut kernel. SG reflects the increase in solids during osmosis, accounting for the transfer of the osmotic agent into the coconut kernel while water is removed. Additionally, the dry basis solid gain rate (DSG) is the solid gain rate at a specific osmosis time divided by the initial solid content, providing a measure of the increase in solids relative to the dry matter content of the coconut kernel. These indicators are essential for evaluating the effectiveness of mass transfer by osmotic dehydration. We investigated the impact of sucrose on the water loss rate, solid gain rate, dehydration efficiency, and dry basis solid gain rate of coconut kernel under both solid and liquid osmosis conditions. The results of our analysis are presented in Figure 1.

Figure 1a clearly demonstrates that the water loss rate during SSD is significantly higher than LOD, particularly at the initial stages of the process. This can be attributed to the higher osmotic pressure in SSD compared to LOD. At the beginning of osmosis, the substantial osmotic pressure difference between the sucrose or sucrose solution and the coconut kernel cells leads to a rapid increase in the water loss rate. As osmosis progresses, the concentration of the sucrose solution gradually decreases, which in turn reduces the osmotic pressure difference between the inside and outside of the coconut kernel cubes. Consequently, the increase in the water loss rate slows down and eventually stabilizes in the later stages of osmosis [8,17]. During the SSD process, the flow of water from the coconut cubes leads to sucrose dissolving, resulting in a gradual decrease in the mass ratio of sucrose to coconut cubes, thereby altering the dehydration rate. In the early stage of SSD, it can be regarded as the osmosis of a saturated sugar solution; hence, the water loss rate in SSD is significantly higher than that of LOD.

Fick’s second law of diffusion describes the diffusion rate using a diffusion coefficient, which is defined as the rate at which a substance passes through a unit cross-sectional area, divided by the concentration gradient across that cross-section. By fitting the experimental data to this equation, the diffusion coefficient can be calculated to evaluate the substance’s diffusion rate [10]. This study employs the Fick equation to compare water diffusion in coconut kernels during solid and liquid osmosis. The Fick equation effectively simulates the changes in water loss rate and osmosis time for the coconut kernel. The correlation coefficients for the fitted equations in solid and liquid osmosis are 0.9599 and 0.9713, respectively, indicating a good fit. The slope of the fitted equation represents the water diffusion coefficient of the coconut kernel under different osmosis methods. The results indicate that the water diffusion coefficients for SSD and LOD coconut kernels are 9.0396 h^−1/2^ and 2.9940 h^−1/2^, respectively. This signifies that the water diffusion coefficient for SSD coconut kernel is 201.92% higher compared to LOD.

#### 3.1.2. Effects of SSD and LOD of Sucrose on Solid Gain Rate

Figure 1b illustrates that the solid gain rate of the coconut kernel in both solid and liquid osmosis initially increases rapidly, then more slowly, and finally stabilizes as osmosis time progresses. During the first 12 h of osmosis, the solid gain rate in solid osmosis rises sharply. After this initial period, the rate of increase begins to decelerate. This deceleration in the later stages is attributed to continuous dehydration, the complete dissolution of sucrose, and a decrease in sucrose concentration, which reduces osmotic pressure. In the early stages of osmosis, the rapid water loss and solute absorption cause changes in the structure of the coconut kernel’s surface layer, compacting it and increasing resistance to the mass transfer of water and solutes, which subsequently slows down the solid gain rate. The difference in solid gain rate between the two osmosis methods is negligible during the first 2 h. However, after 2 h, the solid gain rate in solid osmosis becomes significantly higher than in liquid osmosis. This difference arises because, within the first 2 h, the sucrose in solid osmosis has not completely dissolved in the water exuded from the coconut kernel, leading to a lower sugar content infiltrating the kernel. As osmosis time extends, the sucrose in solid osmosis dissolves in the exuded water, resulting in a higher sucrose concentration compared to liquid osmosis, and consequently a higher solid gain rate in solid osmosis [24].

Figure 1c shows that the dry basis solid gain rate of the coconut kernel in both solid and liquid osmosis initially increases rapidly, then slows down, and eventually stabilizes as osmosis time progresses. Additionally, at any given osmosis time, the dry basis solid gain rate in solid osmosis is higher than that in liquid osmosis. This observation is consistent with the findings reported by Nićetin et al. [25]. Furthermore, it can be observed from Figure 1c that within the first 10 h of the osmosis process, the dry basis solid increase rate changes rapidly. During the initial 5 h, the dry basis solid increase rate is less than 30%, indicating that the coconut cubes have absorbed a small amount of sucrose, which has not significantly altered the texture and shape of the coconut cubes.

#### 3.1.3. Effects of SSD and LOD of Sucrose on Dehydration Efficiency of Coconut Kernel

Figure 1d shows that the dehydration efficiency of the coconut kernel in both solid and liquid osmosis gradually decreases as osmosis time increases. This decrease is primarily due to the continuous loss of water from the coconut kernel and the subsequent reduction in the concentration of the sugar solution, which lowers the osmotic pressure gradient between the interior and exterior of the coconut cells, leading to a decline in dehydration efficiency. These experimental results align with the findings of Wang [26]. Furthermore, at any given osmosis time, the dehydration efficiency of the coconut kernel in solid osmosis is higher than in liquid osmosis, underscoring the advantages of solid osmosis. The solid-state osmosis process shows the advantages of shorter dehydration times and higher dehydration efficiency.

#### 3.1.4. Effects of SSD and LOD of Sucrose on Mass Transfer Coefficient and Osmotic Equilibrium Point

**Figure 2:**
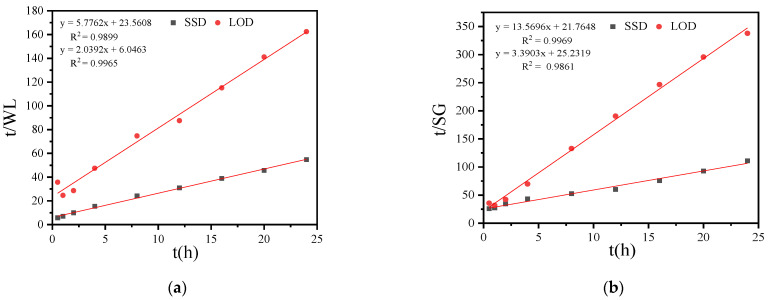
Relationship between t/WL and time t (**a**), relationship between t/SG and time t (**b**) during SSD and LOD of coconut kernel.

The Azuara model is utilized to fit the osmosis process, enabling the determination of the mass transfer coefficient and the osmotic equilibrium point. This model complements the Fick model by providing a more comprehensive explanation of diffusion, mass transfer, and the osmotic equilibrium point during the osmosis process, thereby offering a more detailed description of osmosis kinetics [27], with the results presented in Figure 2a.

Figure 2a demonstrates that the Azuara model effectively simulates the variation in the water loss rate of the coconut kernel over time during osmosis. The slope of the line represents the reciprocal of the equilibrium water loss rate of the coconut kernel under different osmosis methods, while the intercept represents the reciprocal of the product of the water mass transfer coefficient and the equilibrium water loss rate of the coconut kernel under these methods [28].By establishing the fitting equations and performing the corresponding calculations, the equilibrium water loss rates (WL_∞_) of the coconut kernel in SSD and LOD are found to be 49.04% and 17.31%, respectively. The higher equilibrium water loss rate observed in SSD is likely due to the greater osmotic pressure in SSD compared to LOD, which results in a faster water loss rate in SSD.

Figure 2b shows that the Azuara model effectively simulates the variation in the solid gain rate of the coconut kernel over time during osmosis. The slope of the line represents the reciprocal of the equilibrium solid gain rate under different osmosis methods [29], while the intercept represents the reciprocal of the product of the solid mass transfer coefficient and the equilibrium solid gain rate under these methods. By establishing the fitting equations and performing the necessary calculations, the equilibrium solid gain rates of the coconut kernel in SSD and LOD are determined to be 29.50% and 7.37%, respectively. The higher equilibrium solid gain rate observed in SSD is primarily due to the higher concentration of the sucrose solution in solid osmosis, which facilitates easier diffusion of sucrose into the coconut kernel.

In summary, the mass transfer indicators of the coconut kernel in SSD are significantly higher than in LOD. Therefore, solid osmosis dehydration will be used to treat the coconut kernel in the subsequent study.

### 3.2. Effects of Solid Osmosis of Sucrose and Sorbitol on Dehydration and Mass Transfer of Coconut Kernel

#### 3.2.1. Effects of Solid Osmosis of Sucrose and Sorbitol on the Water Loss Rate of Coconut Kernel

**Figure 3:**
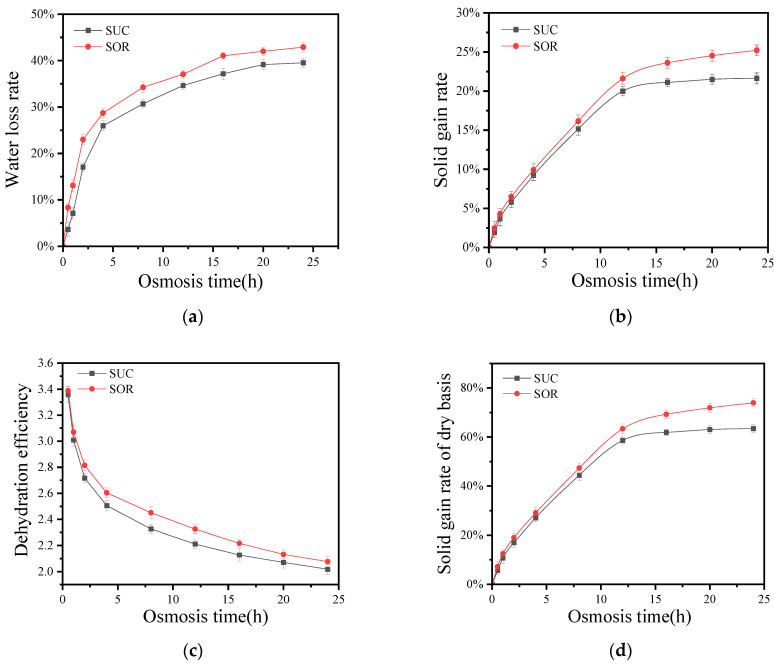
Changes of water loss rate (**a**), solid gain rate (**b**), dehydration efficiency (**c**), and dry basis solid gain rate (**d**) of coconut meat under the condition of sucrose and sorbitol solid infiltration.

Sucrose is the most commonly used osmotic agent, but excessive consumption can lead to dental caries in children and pose significant health risks for individuals with diabetes and obesity. Sorbitol, on the other hand, offers a cool sweetness at about 60% of the sweetness of sucrose and is not metabolized by certain bacteria. This makes it a valuable ingredient in the production of sugar-free candies and various anti-caries foods [8,30]. In Section 3.1 of the study, the dehydration efficiency of SSD is better than that of LOD. Further, the osmotic dehydration mass transfer effect of sucrose and sorbitol in SSD is investigated. Figure 3 illustrates the water loss rate (a), solid gain rate (b), dehydration efficiency (c), and dry basis solid gain rate (d) during the solid osmosis process using both sucrose and sorbitol.

With the increase in osmosis time, due to the osmotic pressure between the osmotic agent and coconut cells, the coconut kernel continuously loses water. As the coconut kernel loses water, the osmotic pressure between the osmotic agent and coconut cells gradually decreases. Therefore, as shown in Figure 3a, the water loss rate of coconut kernel in sucrose osmosis (SUC) and sorbitol osmosis (SOR) first increases rapidly, then slowly, and finally stabilizes.

However, at the same osmosis time, the water loss rate of SOR is higher than that of SUC. This may be because the molecular weight of sorbitol is 182.17, whereas the molecular weight of sucrose is 342.3, resulting in a higher osmotic pressure for sorbitol.

The Fick equation effectively simulates the variation in the water loss rate of the coconut kernel over time for both sucrose and sorbitol osmosis. By establishing the fitting equations and analyzing their expressions, the water diffusion coefficients of the coconut kernel in sucrose (SUC) and sorbitol (SOR) osmosis are determined to be 8.6771 h^−1/2^ and 8.7323 h^−1/2^, respectively. It is observed that the water diffusion coefficient for SOR is slightly higher, with an increase of 0.64%, mainly because, at the same osmosis time, the water loss rate for SOR is higher than that for SUC. This finding is consistent with the results reported by Mustafa et al. [31].

#### 3.2.2. Effects of Solid Osmosis of Sucrose and Sorbitol on the Solid Gain Rate of Coconut Kernel

Figure 3b shows that the solid gain rate of the coconut kernel in both sucrose osmosis and sorbitol osmosis initially increases rapidly, then slows down, and eventually stabilizes as osmosis time progresses. This pattern is primarily due to the continuous dehydration mass transfer until osmotic equilibrium is reached. As osmosis time increases, the osmotic pressure between the osmotic agent and the coconut cells gradually decreases, causing the rate of solid gain to progressively slow down [32].

Figure 3b shows that the solid gain rate of the coconut kernel in both sucrose (SUC) and sorbitol (SOR) osmosis increases rapidly during the first 12 h. This characteristic can be advantageous in industrial production for the rapid incorporation of solid osmotic agents in the processing of fruits and vegetables [20]. After the initial 12 h, the solid gain rate increases more slowly until it stabilizes. This slower phase can be effectively utilized for the precise quantification of nutrient enhancement through osmosis [11].

Additionally, at the same osmosis time, the solid gain rate for sorbitol (SOR) is higher than that for sucrose (SUC). This difference can be attributed to the smaller molecular weight of sorbitol compared to sucrose, which allows sorbitol to penetrate the coconut kernel more easily, resulting in a higher solid gain rate. The Fick equation effectively simulates the variation in the solid gain rate of the coconut kernel over time during osmosis.

#### 3.2.3. Effects of SSD of Sucrose and Sorbitol on Dehydration Efficiency and Dry Basis Solid Gain Rate

Figure 3c shows that the dehydration efficiency of the coconut kernel in both sucrose (SUC) and sorbitol (SOR) decreases rapidly during the first 8 h, after which the rate of decrease slows down. This trend is primarily due to the continuous water loss from the coconut kernel, which gradually reduces the osmotic pressure gradient between the inside and outside of the coconut cells, leading to a decrease in dehydration efficiency over time [33]. Additionally, at any given osmosis time, the dehydration efficiency of SOR is higher than that of SUC.

Dehydration efficiency is a crucial metric for evaluating the speed of dehydration, but during the osmosis process, water removal and the increase in dry basis solid content occur simultaneously. Figure 3d illustrates that the dry basis solid gain rate of the coconut kernel in both sucrose (SUC) and sorbitol (SOR) osmosis increases rapidly in the first 12 h, then slows down, and finally stabilizes. This trend aligns with the solid gain rate pattern observed in the solid osmosis of both osmotic agents. At any given osmosis time, the dry basis solid gain rate for SOR is higher than that for SUC. This could be due to sorbitol’s polyhydroxy nature, which allows it to form hydrogen bonds with water molecules, resulting in a strong water-holding capacity [34]. Additionally, driven by the osmotic pressure between the coconut kernel and sorbitol, sorbitol continuously penetrates into the coconut kernel while the kernel loses water. This strong material flow and water loss cause the coconut cell membrane to rupture, reducing the resistance for sorbitol to enter the kernel. Consequently, more sorbitol penetrates into the coconut kernel compared to sucrose, leading to a higher dry basis solid gain rate for SOR.

#### 3.2.4. Effects of SSD of Sucrose and Sorbitol on Mass Transfer Coefficient and Osmotic Equilibrium Point

**Figure 4:**
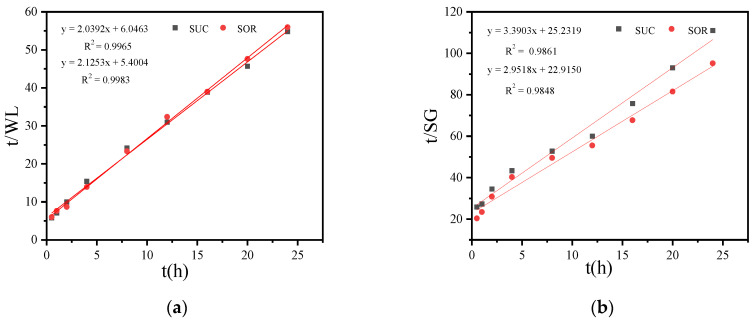
Relationship between t/WL and time t (**a**), relationship between t/SG and time t (**b**) during SSD of coconut kernel using sucrose and sorbitol.

Figure 4a demonstrates that the Azuara equation accurately fits the relationship between t/WL and time t for the coconut kernel. The equilibrium water loss rate for the SOR coconut kernel is higher, likely due to sorbitol’s smaller molecular weight and its stronger water-binding capacity compared to sucrose. This results in higher osmotic pressure and more efficient water loss in the SOR coconut kernel.

Figure 4b shows that the Azuara equation accurately fits the relationship between t/SG and time t or the coconut kernel. The equilibrium solid gain rate for the SOR coconut kernel is higher, which can be attributed to sorbitol’s smaller molecular weight compared to sucrose. This smaller molecular size allows sorbitol to penetrate the coconut kernel more easily.

## 4. Conclusions

The effects of solid osmosis and liquid osmosis, as well as sucrose and sorbitol, on the mass transfer of tender coconut kernels were investigated. The solid osmosis of tender coconut kernels had a higher equilibrium water loss rate and dehydration efficiency, and the dehydration and mass transfer effects of sorbitol were better than those of sucrose. This study may provide a reference for the theoretical study of osmosis, dehydration, and mass transfer of tender coconut kernel and also provide technical support for the development and utilization of tender coconut kernel. However, the effects of osmotic dehydration on the chemical, organoleptic, textural, and sensory properties of tender coconut kernels have not yet been studied. This will be the focal point of our subsequent research.

## Data Availability

The original contributions presented in the study are included in the article, further inquiries can be directed to the corresponding author.
